# Association between General Movements Assessment and Later Motor Delay (excluding Cerebral Palsy) in Low-Birth-Weight Infants

**DOI:** 10.3390/brainsci12060686

**Published:** 2022-05-24

**Authors:** Hirotaka Gima, Tomohiko Nakamura

**Affiliations:** 1 Department of Physical Therapy, Graduate School of Human Health Sciences, Tokyo Metropolitan University, 7-2-10 Higashi-Ogu, Arakawa-ku, Tokyo 116-8551, Japan; 2 Department of Neonatology, Nagano Children’s Hospital, 3100, Toyoshina, Azumino City, Nagano 399-8288, Japan; tomohiko-nakamura@nkodomo-hsp.jp

**Keywords:** general movements, developmental coordination disorder, low-birth-weight infant

## Abstract

The general movements (GMs) assessment is useful for the prediction of cerebral palsy (CP) and other developmental disorders. Developmental coordination disorder (DCD) is highly prevalent in low-birth-weight (LBW) infants. We investigated the association between aberrant GMs during early infancy and later motor development in LBW infants. The study included infants who fulfilled the following criteria: GMs assessed at 9–20 weeks post-term age; developmental quotient (DQ) assessed at 3 years of age using the Kyoto Scale; intelligence quotient (IQ) assessed at 6 years of age. Participants with normal IQs at 6 years of age without a diagnosis of CP (14 males and 37 females, 23–36 weeks gestation with birth weights of 492–1498 g) were categorized into normal (*n* = 39) and aberrant (*n* = 12) groups based on GMs assessment; DQ was compared between the groups. We investigated the items in the DQ assessment and found that the infants in the aberrant group were more frequently unable to perform. Infants in the aberrant group showed a significantly lower DQ in the ‘postural-motor domain’, and were more frequently unable to ‘climb the stairs with alternating legs’ and ‘Jump from a 15–20 cm platform’. This study highlights that GMs aberrancy in early infancy is associated with a delayed gross motor development, even in children with a typical development. The GMs assessment may be useful for the prediction of DCD.

## 1. Introduction

Medical technology has significantly improved the survival rate of low-birth-weight infants, but they are at an increased risk of neurodevelopmental abnormalities [1,2]. Previous studies have reported long-term neurodevelopmental morbidity, especially cerebral palsy (CP), intellectual disability (ID), and autism spectrum disorder (ASD), in very-low-birth-weight infants [3,4,5,6,7,8,9]. In addition, preterm birth and low birth weight have recently been reported as risk factors for later developmental coordination disorder (DCD) [10,11,12,13]. DCD is the most common motor disorder in school-aged children and is characterized by clumsiness in fine (handwriting and shoelace tying) and gross (playing sports and changing clothes) motor skills [14,15]. In addition to affecting daily life performance, DCD in childhood has been reported to have psychological implications, such as a reduced self-esteem and increased risk of anxiety and depression [16,17,18]. Furthermore, it has been reported that 50–70% of children with DCD have motor difficulties that persist through adolescence and adulthood [14,15], making early detection and effective intervention challenging.

Previous studies have reported that the qualitative assessment of general movements (GMs) can be used as a diagnostic tool for predicting neurodevelopmental disorders, especially CP [19,20]. Other assessment methods, such as the Hammersmith Infant Neurological Examination, the Neurobehavioral Assessment of the Preterm Infant, the Bayley Scales of Infant and Toddler Development, Third Edition, and the Neonatal Behavioural Assessment Scale, are used in clinical practice and research as early predictors of motor dysfunction. However, the GMs assessment is reported to be more accurate in predicting a wide range of motor dysfunctions from mild to severe [21]. The GMs were defined by Prechtl et al. [22]. GMs emerge in the foetus at 8–10 weeks of gestational age, and continue to be present until 5 months of life, changing their movement properties from writhing movements to fidgety movements (FMs) within the second month of post-term age [23]. FMs are characterized by small continual movements of moderate speed and a variable acceleration of the neck, trunk, and limbs in all directions [22,23]. Qualitative aberrations in FMs have been reported to be associated with later motor dysfunction. The absence of FMs, as one of the aberrant GMs, is characterized by only sporadically present or altogether absent FMs, and has been reported to be strongly associated with later CP [24,25,26,27,28,29]. In addition, several studies have reported that the assessment of FMs is useful not only for the early prediction of CP, but also for the prediction of motor delays without CP [21]. Abnormal FMs, as one of the aberrant GMs, exaggerated in amplitude, speed, and jerkiness, have been reported to be later associated with minor neurological dysfunction (MND) [30,31,32].

Recently, it has been hypothesized that CP and DCD have similar causal pathways and may fall on a spectrum of movement disorders rather than discrete categories [33,34,35]. Especially in preterm infants, there is some evidence that risk factors for both CP and DCD show a considerable overlap, and that the neural structure, at both macro and micro levels, shows similarities [35]. Furthermore, there are reviews having reported the increased occurrence of DCD or motor impairment in preterm children who do not develop CP [10,36]. From the hypothesis of a continuum between CP and DCD, the characteristics of GMs in early infancy have the potential to be associated with later DCD.

This study aimed to investigate the association between aberrations in FMs at 9–20 weeks of post-term age and delayed motor development in low-birth-weight infants who did not later develop CP, ID, and ASD. In particular, we examined motor function at age 3 in subjects who showed the absence of FMs, but did not have a CP outcome. We further examined the possible relationship between DCD and FMs, which has been suggested to be in continuum with CP.

## 2. Materials and Methods

### 2.1. Participants

**Figure 1:**
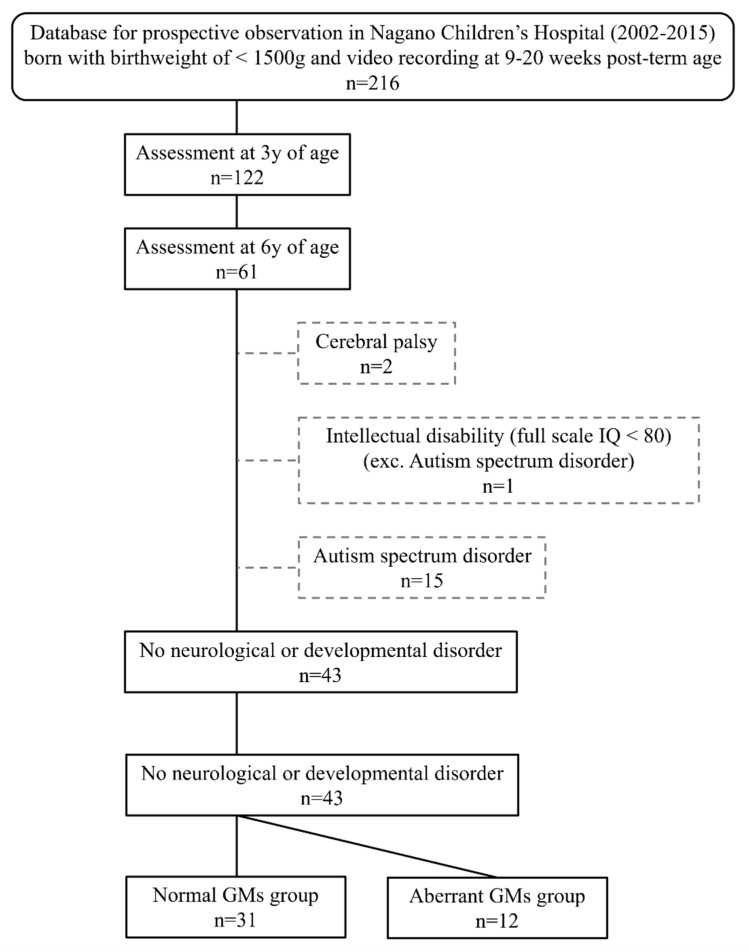
Flow diagram of participant enrolment.

This study was conducted as part of a prospective observational study of infants born between 2002 and 2015 in the neonatal intensive care unit of Nagano Children’s Hospital, Nagano, Japan. In Japan, the recommended follow-up system for low-birth-weight infants is the protocol up to the age of 9 years old, which is now used in many hospitals. The protocol recommends follow-up check-ups at 1.5 years and 3 years for developmental assessment using the Kyoto Scale [37], and at 6 and 9 years using the Wechsler Intelligence Scale for Children (WISC). Figure 1 shows a flow diagram of participant enrolment. The study included low-birth-weight infants who fulfilled the following criteria: (1) GMs assessed at early infancy (9–20 weeks post-term age); (2) developmental quotient (DQ) assessed at approximately 3 years of age; (3) intelligence quotient (IQ) assessed at approximately six years of age. In this study, participants were selected strictly on the basis of developmental assessment and diagnosis at an age of 6 years old. Low birth-weight is defined as a birth weight < 2500 g. In this study, we included extremely low birth-weight (birth weight < 1500 g) among low-birth-weight infants. A total of 61 children fulfilled these criteria. We then excluded participants with a diagnosis of CP (*n* = 2), ID (*n* = 1), or ASD (*n* = 15) at the age of 6 years from the subject group; finally, 43 children were included in the study. Video data of these infants were used to assess GMs. Participants with no neurological developmental disorder were categorized into normal FMs and aberrant FMs groups based on GMs assessment.

The characteristics of the normal and aberrant FMs groups are listed in Table 1. There were 31 infants in the normal FMs group (12 males, 27 females; mean gestational age ± standard deviation, 28 weeks 6 days ± 3 weeks 0 days; mean birth weight ± standard deviation, 1050.1 ± 278.1 g) and 12 infants in the aberrant FMs group (2 males, 10 females; mean gestational age ± standard deviation, 27 weeks 5 days ± 2 weeks 6 days; mean birth weight ± standard deviation, 826.8 ± 233.8 g). No significant between-group differences were found in sex (*p* = 0.28), gestational age (*p* = 0.28), duration of hospital stay (*p* = 0.06), age at the recording of spontaneous movements (*p* = 0.62), age at DQ assessment (*p* = 0.11), and age at IQ assessment (*p* = 0.57). The birth weight (*p* = 0.02) and Apgar score at 5 min (0.04) were significantly lower in the aberrant FMs group than in the normal FMs group. We performed an intergroup comparison of the DQ. Additionally, we explored which items of the DQ assessment infants in the aberrant group were more frequently unable to perform.

### 2.2. Assessment of GMs

The GMs of the infants were video-recorded at the 9–20 weeks post-term age. The mean age of recording was 54 weeks for both the normal FMs and aberrant FMs groups (Table 1). Recordings were obtained using a digital video camera (SONY DCR-TRV20 or DCR-PC350; Sony Corporation, Tokyo, Japan; sampling frequency, 30 Hz). During the recordings, the infants were placed in the supine position and GMs were recorded during the awake, active, and noncrying states. We selected a 1 min to 5 min period, depending on the infant’s state, during which continuous movement occurred, which was used for assessment. We performed visual Gestalt perception using GMs assessment [23] and classified them into two types of GMs (normal or aberrant FMs). In this study, the absence of FMs and abnormal FMs in the definition of GMs assessment were classified as aberrant FMs (Table 2). As a result, aberrant FMs were the absence of FMs in all cases. For consistency, assessments of GMs were performed by a single physical therapist who had GMs assessment basic course certification (H.G.). The assessor had more than 15 years of experience in physical therapy of low-birth-weight infants and neonates, and was involved in the research on GMs assessment. The assessor was blinded to the participant characteristics (sex, birth weight, and gestational age).

### 2.3. Developmental Assessment at 3 and 6 Years of Age

The DQ at 3 years of age was assessed using the Kyoto Scale [37], which was created by the Kyoto City Children’s Hospital in 1980. It is an individualized, face-to-face test based on observations of natural actions when assessment tools are presented to children. The scale examines children’s general developmental progress and delays in the following three domains: postural–motor, cognitive–adaptive, and language–social domains. In each of these three domains, the developmental age for each domain and the total developmental age are divided by the child’s chronological age and multiplied by 100 to yield four developmental quotients (including the total DQ). Furthermore, in this study, we selected the following 17 items in the PM and CA domains that may have reflected gross motor and fine motor functions: (1) jump up with both feet in two or three repetitions, (2) jump at least two to three steps forward on one leg, (3) climb up and down the stairs by grasping the handrail (both feet may be placed together on each step), (4) climb the stairs with alternating legs, (5) jump from a 15–20 cm platform, (6) stack five building blocks, (7) stack blocks to imitate a gate, (8) insert square plates into a hole, (9) fit the board into the frame, (10) fold paper (origami), (11) hold boxes in hand and compare the weights, (12) draw in a circular or spiral pattern, (13) draw a horizontal line, (14) draw a vertical line, (15) draw a cross, (16) draw a circle, and (17) draw the necessary parts of an unfinished portrait. Then, we calculated the percentage of accomplishments for each task.

IQ at 6 years of age was assessed using the WISC (3rd or 4th edition) and examined by clinical psychologists to assess verbal, performance, and full-scale IQs. The developmental function was classified as delayed for a full-scale IQ < 70. Furthermore, we obtained information regarding these participants from their parents and kindergarten teachers when they were 6 years old. Diagnoses of CP and ASD were determined by medical doctors specializing in paediatric neurology. To diagnose CP, we adopted the definition provided by the Ministry of Health and Welfare, Japan. In addition, ASD was diagnosed in accordance with the criteria of the Diagnostic and Statistical Manual of Mental Disorders (DSM) (4th edition).

### 2.4. Data Analysis

The Statistical Package for the Social Sciences version 24 (SPSS, IBM Japan Inc., Tokyo, Japan) was used for the statistical analysis. The statistical analysis method for each data set was chosen based on the Shapiro–Wilk test of normality. Regarding participants’ information, we used Fisher’s exact test for intergroup comparisons of sex. We used the Student’s t-test for gestational age, birth weight, and age at recording spontaneous movements. We used the Mann–Whitney U test for intergroup comparisons of Apgar score at 5 min, duration of hospital stay, age at DQ assessment, and age at IQ assessment. The cut-off level for statistical significance was set at *p* = 0.05.

For the intergroup comparison of the DQ at 3 years of age, we conducted the Mann–Whitney U test, because the DQ of each domain was not normally distributed based on the Shapiro–Wilk test. Statistical significance was defined as *p* < 0.05.

For each item in the DQ assessment, we used Fisher’s exact test to explore which items were more frequently not performed by the aberrant group. We corrected the *p*-values using the Benjamini–Hochberg method to account for the effect of false positives due to multiple testing [38]. The false discovery rate was set to 0.05 to carry out an exploratory test to find the important items.

## 3. Results

### 3.1. DQ at 3 Years of Age

**Figure 2:**
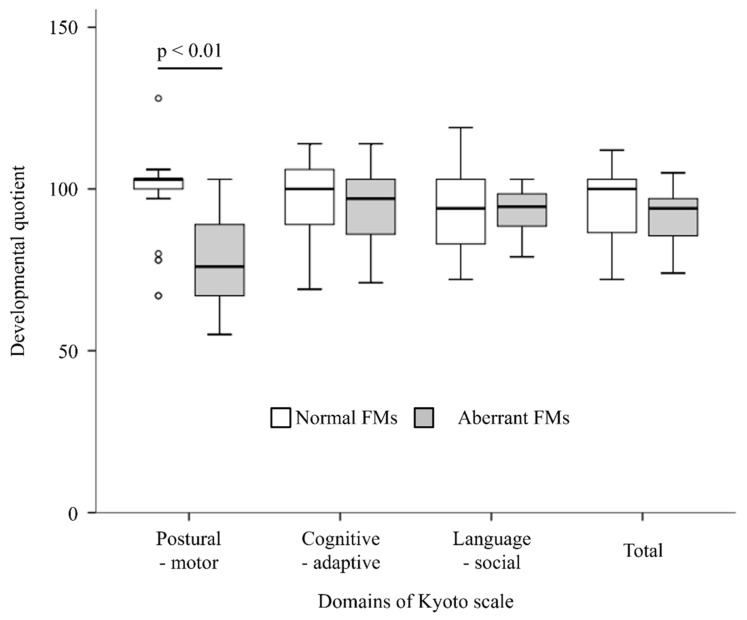
The DQ values at 3 years of age for the normal and aberrant groups.

The DQ values at 3 years of age for the normal and aberrant groups are shown in Figure 2. The DQ of the postural–motor domain was significantly lower in the aberrant FMs group than in the normal FMs group (*p* < 0.01). No significant between-group differences were found in the cognitive–adaptive and language–social domains and total DQ.

### 3.2. Items Related to Motor Function in the DQ Assessment

The percentages of items related to motor function in the DQ assessment are shown in Table 3. Participants in the aberrant FMs group were significantly more frequently unable to ‘climb the stairs with alternating legs’ (41.7%; *n* = 5) and ‘jump from a 15–20 cm platform’ (50.0%; *n* = 6) than those in the normal FMs group (*p* < 0.01). No significant between-group differences were observed for the other items. There was no difference between the aberrant FMs and normal FMs groups in the percentage of the achievement of test items requiring fine motor skills, such as ‘fold a paper (origami)’ and ‘draw a circle’.

## 4. Discussion

In this study, we investigated the association between aberrations in GMs in early infancy and later motor development, especially in low-birth-weight infants who did not later develop CP, ID, and ASD. The major findings of the present study were as follows: (1) the DQ of the postural–motor domain was lower in the aberrant FMs group than in the normal FMs group, and (2) the percentage of the achievement of the test items related to gross motor function was lower in the aberrant FMs group than in the normal FMs group.

In this study, we selected participants who were considered to have a typical development in IQ at 6 years of age, but 12 infants showed aberrant FMs (absence of FMs) in the GMs assessment in early infancy. The assessment of FMs has been increasingly used to predict CP in clinical practice and research [19,20]. The absence of FMs has especially been reported to be strongly associated with later CP [24,25,26,27,28,29]. In addition, several studies have reported that abnormal FMs are useful for the early prediction of motor delays without CP, such as MND [30,31,32]. Einspieler et al. reported that abnormal FMs were related to dysfunction in the fine manipulative ability at puberty [31]. Furthermore, Nakajima et al. [32] and Bruggink et al. [30] examined the association between FMs and later motor function in low-birth-weight infants, and reported that infants who showed abnormal FMs had a higher rate of later MND. These reports suggest that the dysfunction of coordination and balance, fine manipulation, and posture and muscle tone due to MND may be detected early by the evaluation of abnormal FMs. On the other hand, Spittle et al. [39] and Peyton et al. [40] also reported an association between aberrant FMs and later delayed motor development in low-birth-weight infants, but the aberrant FMs group subjects in these studies was mostly absence of FMs. In our results, the DQ of the postural–motor domain at 3 years of age was significantly lower in the aberrant FMs group than in the normal FMs group. This result supports the reports by Spittle et al. and Peyton et al., and suggests that the absence of FMs in early infancy may also predict delayed motor function at 3 years of age in low-birth-weight infants. Furthermore, our results showed a lower percentage of achievement for test items of the DQ assessment ‘climb the stairs with alternating legs’ and ‘jump from a 15–20 cm platform’ in participants who showed aberrant FMs in early infancy. These items are related to gross motor function. There was no difference between the aberrant FMs and normal FMs groups in the percentage of the achievement of test items requiring fine motor skills, such as ‘fold a paper (origami)’ and ‘draw a circle’, suggesting that aberrant FMs in early infancy may later predict a delay in gross motor function in particular. In view of these reports and results, aberrant FMs may be an early sign of later motor dysfunction, whether they be abnormal FMs or the absence of FMs, but each may reflect different causes of motor dysfunction.

In contrast, our results showed that the birth weight and Apgar score at 5 min were lower in the aberrant FMs group than in the normal FMs group. This result was similar to our previous study, in which we examined the qualitative characteristics of FMs in a larger number of subjects [41]. A low birth weight and Apgar score at 5 min were shown to be high-risk factors for later CP and DCD, suggesting that aberrant FMs are more frequently present in infants who are at a higher risk of dysfunction of neurological development. Recently, it has been hypothesized that CP and DCD have similar causal pathways and may fall on a continuum of movement disorder categories [33,34,35]. Pearsall-Jones et al. reported that children who met the criteria for DCD experienced perinatal oxygen perfusion problems, and suggested that DCD and CP have similar causal pathways [42]. In addition, de Kieviet et al. showed that large reductions in the fractional anisotropy of the corticospinal tract were present in very preterm infants, particularly those with a research diagnosis of DCD, and suggested that this result provided clear evidence that reduced values of fractional anisotropy strongly underpin motor impairment and DCD in very preterm children at school age [43]. If a similar causation (aetiology) in DCD and CP is strongly supported, the GMs assessment may also be useful in the early detection of DCD. Furthermore, preterm birth and a low birth weight have recently been reported as risk factors for later DCD [10,11,12,13], suggesting that the GMs assessment may be particularly associated with DCD caused by preterm birth and/or a low birth weight.

The limitations of this study must be considered. First, it is unknown how many children with DCD were included in the study. Fourteen children with a low DQ in the postural–motor domain were included; however, a low DQ in the postural–motor domain is not directly related to the diagnosis of DCD. The use of the Movement Assessment Battery for Children, Second Edition (MABC2), is recommended as the gold standard for the assessment and diagnosis of DCD. In future research, MABC2 should be additionally assessed, and its relevance to the GMs assessment should be studied in detail. Second, for consistency, the GMs assessment was performed by one assessor. In clinical practice, it is important to have more than three assessors to ensure a reliable evaluation. These are important issues worthy of future investigation. Therefore, we would like to conduct further studies using larger sample sizes to investigate the association between the diagnosis and severity of DCD and the characteristics of GMs in early infancy.

## 5. Conclusions

This study highlighted that aberrant FMs in early infancy were associated with delayed gross motor development at 3 years of age, even in children who showed typical development at 6 years of age. DCD is defined as a disorder of fine and gross motor skills, and has been suggested to have causal pathways similar to those of CP. Therefore, the GMs assessment may be useful for the early prediction of DCD.

## Figures and Tables

**Table 1:** Characteristics of participating infants.

	Normal FMs (*n* = 31)	Aberrant FMs (*n* = 12)
Male/female (*n*) ^a^	12/27	2/10
Gestational age (weeks and days), mean ± SD ^b^	28 w 6 d ± 3 w 0 d	27 w 5 d ± 2 w 6 d
Birth weight (g), mean ± SD ^b^	1050.1 ± 278.1	826.8 ± 233.8 *
Apgar score at 5 min, median (range) ^c^	8 (3–10)	6 (3–9) *
Duration of hospital stay (days), median (range) ^c^	74 (28–194)	108 (28–151)
Age at recording of spontaneous movements (weeks and days), mean ± SD ^b^	54 w 1 d ± 2 w 3 d	54 w 0 d ± 2w 0 d
Age at DQ assessment (years and months), median (range) ^c^	3 y 0 m (2 y 11 m–3 y 2m)	3 y 0 m (2 y 11 m–3 y 1 m)
Age at IQ assessment (years and months), median (range) ^c^	5 y 7 m (5 y 6 m–6 y 2 m)	5 y 7 m (5 y 6 m–5 y 10 m)

FMs, fidgety movements; SD, standard deviation; DQ, developmental quotient; IQ, intelligence quotient; ^a^: Fisher’s exact test; ^b^: *t* test; ^c^: Mann–Whitney U test; *: *p* < 0.05 in comparison with normal FMs group.

**Table 2:** Classification and definition of normal and aberrant FMs.

Classification	Definition
Normal FMs	FMs are small movements of moderate speed and variable acceleration of neck, trunk, and limbs in all directions that are continual in the awake infant during fussing and crying.
Absence of FMs	No FMs can be observed, although other movements can occur.
Abnormal FMs	Abnormal FMs look like normal FMs, but their amplitude, speed, and jerkiness are moderately or greatly exaggerated. Abnormal FMs are rare.

FMs, fidgety movements.

**Table 3:** Percentage of subjects who were able to complete the items.

Test Items Related to Motor Function		Normal FMs (*n* = 31)	Aberrant FMs (*n* = 12)
1	Jump up with both feet in two or three repetitions	93.5	83.3
2	Jump at least two to three steps forward on one leg	3.2	0
3	Climb up and down the stairs by grasping the handrail (both feet may be placed together in each step)	100	100
4	Climb the stairs with alternating legs	93.5	41.7 *
5	Jump from a 15–20 cm platform	90.3	50.0 *
6	Stack five building blocks	100	100
7	Stack blocks to imitate a gate	9.7	0
8	Insert square plates into a hole	100	100
9	Fit the board into the frame	100	100
10	Fold paper (origami)	29.0	33.3
11	Hold boxes in hand and compare the weights	22.6	8.3
12	Draw a circular or spiral pattern	100	100
13	Draw a horizontal line	93.5	91.7
14	Draw a vertical line	96.8	91.7
15	Draw a cross	64.5	66.7
16	Draw a circle	38.7	33.3
17	Draw the necessary parts of an unfinished portrait	9.7	8.3

FMs, fidgety movements; *: *p* < 0.01 in comparison with normal FMs group using the Fisher’s exact test (corrected by the Benjamini–Hochberg method).

## Data Availability

Due to ethical and privacy restrictions, the video data in this study are not publicly available. The other data sets used and/or analysed in this study may be disclosed by the corresponding author upon reasonable request.
